# Fluorescence Quenching of 4-tert-Octylphenol by Room Temperature Ionic Liquids and its Application

**DOI:** 10.1007/s10895-012-1150-1

**Published:** 2012-12-04

**Authors:** Huili Wang, Jingwen Mao, Ailian Duan, Baoguang Che, Wenwei Wang, Meiping Ma, Xuedong Wang

**Affiliations:** 1 School of Life Science, Wenzhou Medical College, University-Town, Wenzhou, Zhejiang China 325035; 2 Department of Environmental Science, Wenzhou Medical College, University-Town, Wenzhou, Zhejiang China 325035

**Keywords:** Ionic liquid, 4-tert-Octylphenol, Fluorescence quenching, Stern-Volmer equation, Quenching mechanism

## Abstract

The interactions between room temperature ionic liquids (RTILs) and weak fluorescent chemicals still remain unclear, which hinders the complete and efficient utilization of these “green” solvents in fluorescent analyses of organic chemicals. Herein, we reported the effects of four RTILs, [C_8_MIM]BF_4,_ [C_14_MIM]BF_4,_ [C_8_MIM]PF_6_ and [C_14_MIM]PF_6_, on fluorescence behavior of 4-tert-octylphenol (4-t-OP). In the fortified concentration range of 0.2–1.0 mM, the quenching effects were increased with increasing concentrations of RTILs. However, no obvious variation of peak shape of 4-t-OP was observed in the quenching process, suggesting no formation of ground-state complex between fluorophores in 4-t-OP and quencher (ionic liquids). As for anion effect, the fluorescence quenching efficiency of 4-t-OP by BF_4_^-^ was greater than PF_6_^-^, but the carbon chain length on the imidazolium ring had no significant relationship with fluorescence intensity of 4-t-OP. Both Ksv values (>1.0 × 10^3^ L/mol.s) and the different temperature effects demonstrated that the quenching of 4-t-OP by four RTILs was the presence of dynamic and static quenching mechanism. The FI of dansyl chloride within [C_8_MIM]BF_4_ increased nearly 5-fold as compared to the control, showing a sensitizing effect on the strong fluorescent chemicals, while a quenching effect on 4-t-OP belonging to weak fluorescent chemicals. The fluorescence-enhanced amplitude of dansyl chloride in [C_8_MIM]PF_6_ was greater than [C_8_MIM]BF_4._ The fluorescence quenching of 4-t-OP by [C_8_MIM]PF_6_ did not belong to FRET phenomenon because of no overlap of emission spectrum of 4-t-OP and absorption spectrum of [C_8_MIM]PF_6_. When 0.6 mM [C_8_MIM]PF_6_ in acetonitrile was used as the solvent, the detection limit of 4-t-OP was 3.7 μg/L, and the linearity range was 0.01–0.8 mg/L (R^2^ = 0.9990). In summary, these results provide a theoretical foundation for the application of RTILs in weak fluorescent chemicals.

## Introduction

Room temperature ionic liquids (RTILs) are organic salts composed entirely of ions, and alternatives to the conventional and environmentally detrimental volatile solvents. They possess several attractive advantages, such as negligible vapor pressure, wide liquid range (from −90 °C to 400 °C), high thermal stability, capacity for dissolving many chemicals, high electrical conductivity, a wide electrochemical window, non-flammable and recyclable nature [1, 2]. These properties make RTILs wide applications as media of material synthesis [3], electrochemical study [4], separation [5] and chemical reaction [6]. In analytical fields, RTILs were used as stationary phase in gas chromatography [7], and as mobile phase additives in capillary electrophoresis and fluorescence analysis [8, 9]. Zou and coworkers found that part of RTILs can sensitize fluorescence of norfloxacin significantly, which was used to develop sensitive spectrometric method for determination of norfloxacin at trace levels [10]. Novel ionic liquid [C_4_MIM][BA] can significantly enhance the fluorescence of Eu^3+^ and Tb^3+^ [11]. However, [C_4_MIM]PF_6_ can quench the fluorescence of 17 β-estradiol, but enhance the fluorescent sensitivity of its derivatives with dansyl chloride [12]. The fluorescence quenching of polycyclic aromatic hydrocarbons by nitromethane was less efficient in [C_4_MIM]PF_6_ than acetonitrile, which resulted from different solvent viscosity [9]. However, the mechanism on interaction between ionic liquids and fluorescent substances still remain unclear, especially for the weak chemicals with the weak fluorophores. These problems hinder the complete and efficient utilization of these “green” solvents in fluorescent analysis of organic chemicals.

4-tert-Octylphenol (4-t-OP) is an endocrine disruptor chemical and is widely used as an antioxidant in plastic and rubber products. It is also an important degradation product of alkyphenol ethoxylates, which are non-ionic surfactants with emulsifying and dispersing actions in the industrial surfactant, detergents, plasticizers and emulsifiers [13]. As a consequence of its large use in our daily life, 4-t-OP is widely distributed in the environment and presents a potential threat to aquatic life and human health, such as affecting the body’s normal endocrine function, immune function, neurological toxicity, teratogenicity and carcinogenicity [14, 15]. Therefore, it is necessary to develop a quick, sensitive and reliable technique to determine trace 4-t-OP residue in each kind of matrix. Fluorescence determination is a direct, simple and quick method for 4-t-OP analysis in environmental matrices [16, 17]. 4-t-OP is a relatively weak fluorescence chemical with the detection limit of 0.04 mg/L by its own molecular fluorescence [18]. Therefore, it is always derivatized by dansyl chloride to achieve a fluorescent detection level of μg/L[12]. Because its residual level is often detected to be at ng/L-μg/L level in environmental water samples [19, 20], the derivatization of 4-t-OP can not still satisfy the requirement for sensitive fluorescence determination. Therefore, it is urgent that the strongly fluorescent sensitizing reagent or medium be developed. In recent years, our research aimed to screen RTILs with characteristics of fluorescence-enhancing or quenching, and further to apply them in trace residual analysis of organic chemicals. The previous study demonstrated that some RTILs have significantly fluorescence-quenching effects on 4-t-OP. Fluorescence quenching is an important technique used to obtain adequate information about structure and dynamics of fluorescence molecules. It is a process, in which fluorescence intensity (FI) of the solute decreases by a variety of molecular interactions such as excited state reactions, molecular rearrangements, energy transfer, ground-state complex formation and collision-quenching [21–23]. So far, no data are available on the quenching mechanism between RTILs and 4-t-OP. Therefore, the present study was aimed to evaluate the effects of RTILs on fluorescent behavior of 4-t-OP, to analyze the quenching mechanism between them, and to provide foundation for the application of RTILs in weak fluorescent chemicals.

## Experimental Section

### Materials

4-Octylphenol (4-t-OP, purity 99.5 %) was purchased from Sigma-Aldrich (St. Louis, MO, USA). Reference compounds of RTILs, 1-octyl-3-methylimidazolium hexafluorophosphate, [C_8_MIM]PF_6_; 1-tetradecyl-3-methylimidazolium hexafluorophosphate, [C_14_MIM]PF_6_; 1-octyl-3-methyl- imidazolium tetrafluoroborate [C_8_MIM]BF_4_; 1-tetradecyl-3-methyl-imidazolium tetrafluoroborate, [C_14_MIM]BF_4_ with stated purities of 99.0%, were all purchased from Shanghai Chengjie Co. Ltd. (Shanghai, China). The four RTILs were used as received without further purification. Ultrapure water used in the study was purified with a Millipore Mill-Q plus system (Bedford, MA, USA). Acetonitrile was spectroscopic grade and was used as received.

### Sample Preparation and Analytical Methods

The stock solutions of 4-t-OP (100 mg/L) and ionic liquid (0.5 mol/L) were prepared in acetonitrile and stored at −4 °C before use. Working solutions were obtained by appropriate dilution of the stock solution with acetonitrile. To investigate the effects of RTILs, precalculated amount of RTILs was directly added to working solution of 4-t-OP, and the concentration of 4-t-OP in the mixture solution was set at 0.3 mg/L. The mixture solution was then transferred into 1 cm^2^ quartz micro-cuvettes at 25, 35 and 45 °C for fluorescence analysis. Fluorescence spectra were recorded using a model RF-5301PC spectrofluorometer (Shimadzu Corporation, Kyoto, Japan) with a 75 W Xenon arc lamp as the excitation source and single-grating monochromator as wavelength selection device with a slit width of 5 nm. All fluorescence spectra were corrected for the solvent blank.

## Results and Discussion

### Effects of Ionic Liquids on FIs of 4-t-OP

The fluorescence spectra of varying 4-t-OP concentrations in acetonitrile were investigated. The excitation and emission maxima of 4-t-OP were set at 227.0 and 302.0 nm, respectively. The FIs of 4-t-OP increased with increasing its concentrations, i.e., when 4-t-OP concentrations ranged from 0.1 to 0.5 mg/L, its FI increased from 179.319 to 750.596 (a.u.). However, with the further increasing concentrations (>0.5 mg/L), FI of 4-t-OP is out of the detectable range, and thus 0.3 mg/L was selected in the subsequent experiment.

**Fig. 1:**
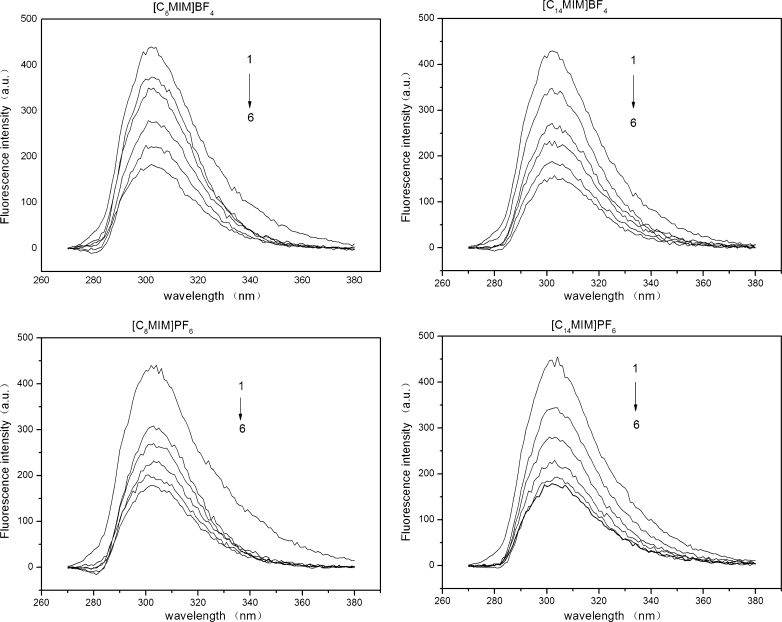
The fluorescence spectra of 4-t-OP in four RTILs with varying concentrations. *1, 0 mM; 2, 0.2 mM; 3, 0.4 mM; 4, 0.6 mM; 5, 0.8 mM; 6, 1.0 mM

The different RTIL concentrations in solvents can lead to a complex interplay due to the different physicochemical properties of solvents (e.g. viscosity, static dielectric constant, refractive index, density, polarizability, etc.), which affects steady-state emission behavior of the analytes. At ambient temperatures, ionic liquids are much more viscous than organic solvents, such as acetonitrile. For example, the viscosity of acetonitrile is 0.37 m Pa s at 20 °C, while that of [C_4_MIM]PF_6_ and [C_4_MIM]BF_4_ is 430 m Pa s and 154 m Pa s, respectively, at the same temperature. Also, the viscosity of ionic liquids, belonging to the same type, was increased with increasing substituent atoms on imidazolium ring [24]. Considering the vast differences among the viscosity values of [CnMIM]PF_6_, [CnMIM]BF_4_ and of the organic solvents. We diluted [C_8_MIM]PF_6_, [C_14_MIM]PF_6_, [C_8_MIM]BF_4_ and [C_14_MIM]BF_4_ to a series of concentrations (0.2, 0.4, 0.6, 0.8 and 1.0 mM) in acetonitrile. The fluorescence spectra of 4-t-OP (0.3 mg/L) are shown in Fig. 1 in the presence of the four RTILs (0–1.0 mM). Obviously, the FI of 4-t-OP was decreased with increasing RTIL concentration, suggesting that RTILs were a quencher in the process of steady-state fluorescence. Also, the shape of fluorescence spectra remained unchanged, and a significant hypsochromic or bathochromic shift of the emission maxima were not observed in the quenching process. [C_8_MIM]PF_6_, [C_14_MIM]PF_6_, [C_8_MIM]BF_4_ and [C_14_MIM]BF_4_ all had a significant quenching effects on fluorescence of 4-t-OP. The quenching effects by ionic liquids can rule out forming possibility of ground-state complex between the fluorophores in 4-t-OP and the quencher (ionic liquids). In addition, these results were in agreement with Fletcher’s observation that the steady-state emission behavior of six polycyclic aromatic hydrocarbons was quenched within [C_4_MIM]PF_6_ [9].

### Effects of Different Anions of RTILs on FIs of 4-t-OP

**Fig. 2:**
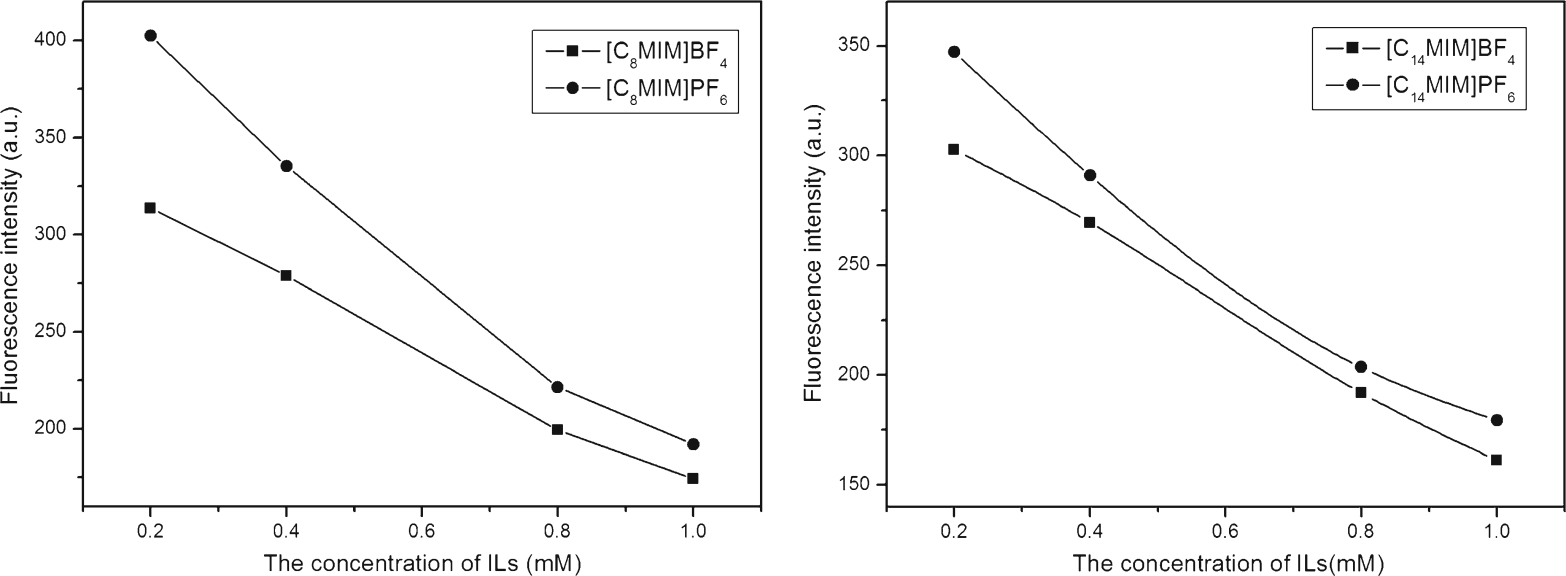
The fluorescence intensity of 4-t-OP in RTILs with different anions

The different cation or anion in RTILs can lead to its different properties, e.g. viscosity, density, melting point and polarity, which affects its fluorescence behavior. The effects of imidazolium ion liquids with BF_4_^-^ or PF_6_^-^ on fluorescence spectra of 4-t-OP are shown in Fig. 2. It was obvious that on addition of [C_n_MIM]BF_4_, the FI of 4-t-OP was decreased more sharply than that of [C_n_MIM]PF_6_. It was reported by Wasserscheid and cowokers that the different anion structures had great impact on the viscosity of RTILs [25]. The higher the anion symmetry, the larger corresponding ionic liquid viscosity was. Compared with BF_4_^−^, the negative charge of PF_6_^−^ anion, due to its octahedral structure, can be well dispersed around the ions to occur more actions in the solvent. In addition to the viscosity differences, the N, F on the anion of ionic liquid can be combined with H atom on cationic imidazolium ring to form hydrogen bond. Because of the strong electrostatic attraction between the anions and cations, the coulomb force of [C_n_MIM]PF_6_ is stronger than that of [C_n_MIM]BF_4_, resulting in the stronger viscosity of PF_6_^−^ ionic liquids [26]. The stronger viscosity can inhibit diffusion of excited state and lead to the reducing of collision probability of fluorophores in micro-environment. Therefore, the FI of 4-t-OP was decreased more weakly due to the greater viscosity of [C_n_MIM]PF_6_.

### Effects of Different Cations of RTILs on FI of 4-t-OP

**Fig. 3:**
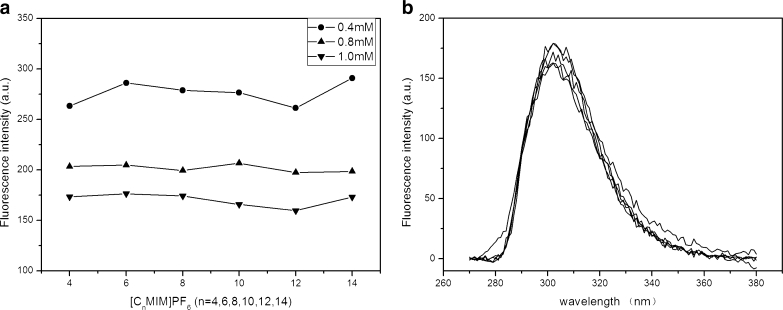
Effects of different carbon chain length. (**a**) The FI of 4-t-OP in [C_n_MIM]PF_6_ with different carbon chain length. (**b**) The fluorescence emission spectra of 4-t-OP in [C_n_MIM]PF_6_ (*n* = 4–14) (The concentration of [C_n_MIM]PF_6_ was 1.0 mM)

[C_n_MIM]PF_6_ with different carbon chain length (*n* = 4, 6, 8, 10, 12, 14) was used to analyze the effects of different cations on the FI of 4-t-OP. As shown in Fig. 3a, no remarkable distinction for the FI of 4-t-OP was observed in the RTIL concentration range of 0.4–1.0 mM with different carbon chain length. Figure 3b shows the fluorescence emission spectra of 4-t-OP in [C_n_MIM]PF_6_ (*n* = 4–14). Obviously, the FI and peak shape of 4-t-OP did not significantly change, and in addition no obvious hysochromic and bathochromic phenomenon occurred for the different chain length treatments. These observations demonstrated that the different carbon chain length of RTILs had no significant effect on fluorescence behavior of 4-t-OP.

### The Fluorescence-quenching Mechanism

**Fig. 4:**
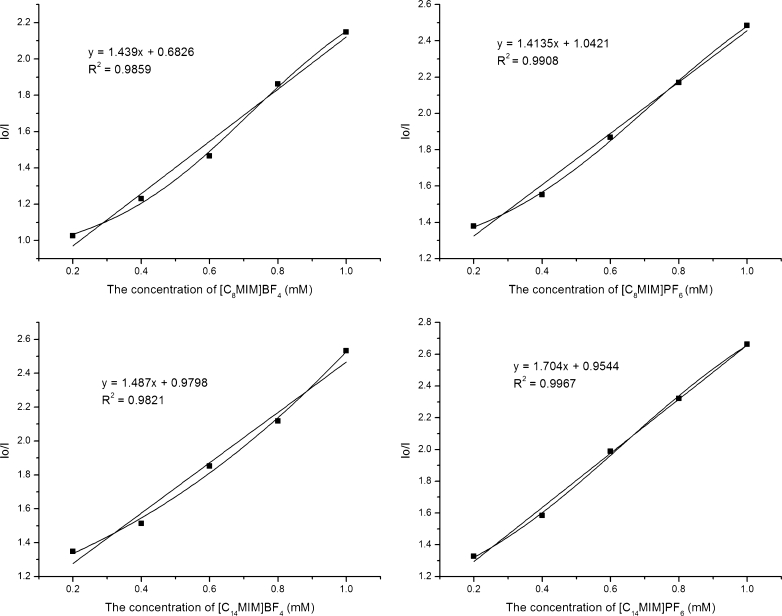
The Stern-Volmer plot for 4-t-OP in different RTILs at 25 °C

Fluorescence quenching is a process that decreases FI of a fluorophore. A variety of molecular interactions can result in fluorescence quenching. There exist two basic types of fluorescence quenching, i.e. one is static (instantaneous) and the other is dynamic (collisional) quenching. Both quenching processes require an interaction between fluorophore and quencher. Static quenching refers to formation of a ground state fluorophore-quencher complex which does not emit a photon. Dynamic quenching refers to formation of an excited state fluorophore-quencher complex [27]. The dynamic process in which quenching mechanism is mainly due to collision is governed by the linear Stern-Volmer (S-V) equation as follows [28]:

$$ {{\mathrm{I}}_0}/\mathrm{I}=1+{{\mathrm{K}}_{\mathrm{SV}}}\left[ \mathrm{Q} \right]=\mathrm{Kq}{\varGamma_0}\left[ \mathrm{Q} \right] $$

where I_0_ and I are steady state fluorescence intensities in the absence and presence of quencher, respectively. Ksv and Kq are the S-V quenching constant and the bimolecular quenching constant, respectively. [Q] is the concentration of quencher. Γ_0_ is the lifetime of fluorophore in the absence of a quencher. Plots of I_0_/I versus concentrations of ionic liquids are shown in Fig. 4. The strong linear correlation coefficients (R^2^ > 0.97) suggest compliance of the S-V equation. The Ksv values were 1.439 × 10^4^ L/mol for [C_8_MIM]BF_4_, 1.487 × 10^4^ L/mol for [C_8_MIM]BF_4_, 1.414 × 10^4^ L/mol for [C_8_MIM]PF_6_ and 1.704 × 10^4^ L/mol for [C_8_MIM]PF_6_, respectively. As reported by Geng et al. [27], the S-V quenching constant for dynamic quenching was less than 1.0 × 10^3^ L/mol, and thus it could be inferred that the 4-t-OP quenching mode by the four ionic liquids was a static quenching mechanism. However, the S-V plots show a slightly positive deviation in Fig. 4, which demonstrated that the quenching of 4-t-OP may be due to the simultaneous presence of dynamic and static quenching. The positive deviation phenomenon for S-V plots has been observed by many researchers [28, 29]. On the contrary, Naik and coworkers found that the S-V plot might show a negative deviation due to different conformations of anthrylyinyl acetate in the ground state [30].

### Effects of the Different Temperatures

**Fig. 5:**
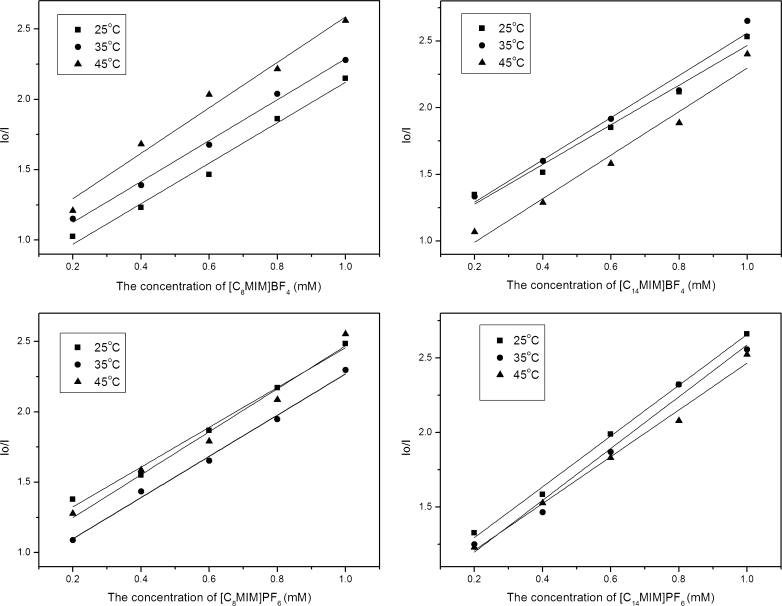
The Stern-Volmer plot for 4-t-OP in different RTILs at varying temperatures

The S-V plots at different temperatures are shown in Fig. 5, and the S-V equation parameters are listed in Table 1. A gradual increasing trend for the quenching constants was observed with increasing temperatures, indicating that the bimolecular quenching reactions were diffusion-limited. The rising temperatures decrease viscosity of the solution, accelerate the molecular motion, and result in increasing molecular diffusion coefficient. The increase in bimolecular quenching constant suggests that the fluorescence-quenching process may be a dynamic quenching mechanism. The conclusion on temperature effect is in concomitant with the above ones on S-V plots, i.e., the simultaneous presence of dynamic and static quenching mechanism.

**Table 1:** The Stern-Volmer equation of 4-t-OP at different temperature

4-t-OP	T/°C	Stern-Volmer equation	R^2^	Ksv(10^4^ L.mol^−1^)
[C_8_MIM]PF_6_	25	y = 1.413x + 1.042	0.991	1.413
	35	y = 1.464x + 0.806	0.995	1.464
	45	y = 1.526x + 0.943	0.979	1.526
[C_14_MIM]PF_6_	25	y = 1.704x + 0.954	0.997	1.704
	35	y = 1.738x + 0.850	0.986	1.738
	45	y = 1.570x + 0.896	0.991	1.570
[C_8_MIM]PF_4_	25	y = 1.439x + 0.683	0.986	1.439
	35	y = 1.453x + 0.835	0.995	1.453
	45	y = 1.615x + 0.971	0.995	1.615
[C_14_MIM]BF_4_	25	y = 1.487x + 0.980	0.982	1.487
	35	y = 1.582x + 0.977	0.977	1.582
	45	y = 1.633x + 0.665	0.974	1.633

### Different Effects of Ionic Liquids on Dansyl Chloride (DNSCl) and 4-t-OP

**Fig. 6:**
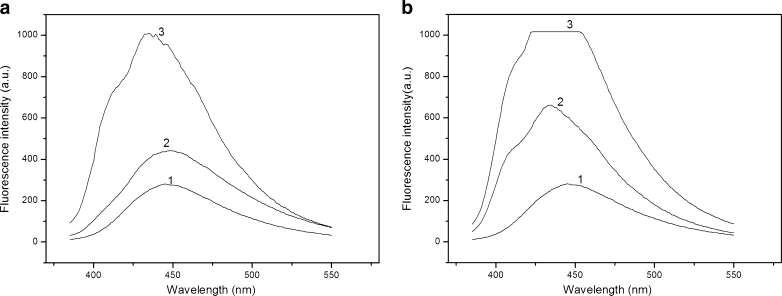
Effects of RTILs on the FI of dansyl chloride. (**a**) [C_8_MIM]BF_4_; (**b**) [C_8_MIM]PF_6_). *1, 0 μL; 2, 100 μL; 3, 300 μL

As reported by Deng and coworkers, interactions of strong or weak fluorescent substances with ionic liquids were obviously different [31]. Wang and coworkers also found that part of ionic liquids had fluorescence-enhancing effect on strong fluorescent chemical (DNSCl), while quenching effect on weak fluorescent chemical (17β-estradiol) [12]. DNSCl is a strong fluorescent substance used as a derivatization reagent to enhance FI of weak fluorescence chemical in fluorescence detection [32, 33]. In this investigation, [C_8_MIM]PF_6_ and [C_8_MIM]BF_4_ were selected to study the different RTIL-mediated effects on the strong (DNSCl) and weak fluorescent chemicals (4-t-OP). Figure 6 shows the FIs of DNSCl in the two RTILs. The more volume (0–300 μL) of ionic liquid was added, the FIs of 4-t-OP were enhanced more. When the volume of [C_8_MIM]BF_4_ was 300 μL, the FI of DNSCl increased nearly 5-fold as compared to the control, and the enhanced amplitude was greater in [C_8_MIM]PF_6_ than [C_8_MIM]BF_4_. However, as discussed above, [C_8_MIM]PF_6_ and [C_8_MIM]BF_4_ had obvious quenching effects on 4-t-OP. The different RTIL-mediated effects on strong or weak fluorescent chemicals were also reported by Wang and coworkers, who found an obvious quenching effect on 17β-estradiol in RTILs [12]. But further research is required to analyze for the mechanism of RTIL-mediated effects on chemicals with strong or weak fluorophores.

### Fluorescence Quenching of 4-t-OP and Fluorescence Resonance Energy Transfer (FRET)

**Fig. 7:**
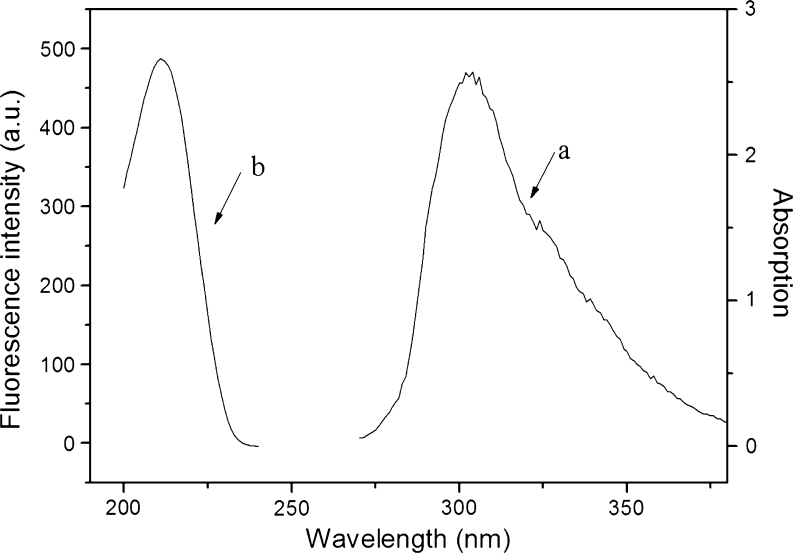
The emission spectra of 4-t-OP (**a**) and absorption spectra of [C_8_MIM]PF_6_ (**b**)

FRET is a photophysical process and occurs when the electronic excitation energy of a donor chromophore is transferred to an acceptor molecule nearby (10–90*Å*) via a dipole–dipole interaction between the donor-acceptor pair [34]. According to the Forster’s theory, the rate of energy transfer is based on an overlap of emission spectrum of the donor and absorption spectrum of the acceptor, relative orientations and distance of the donor and acceptor transition dipoles, and fluorescence quantum yield of the donor. Therefore, FRET process seems to be more efficient when there is an appreciable overlap between the emission spectrum of the donor and the absorption spectrum of the acceptor [35]. As discussed above, the FI of 4-t-OP was decreased with the addition of RTILs. Therefore, it is necessary to judge whether FRET occurs between RTILs and 4-t-OP in the quenching process. Figure 7 shows the emission spectra of 4-t-OP and absorption spectra of [C_8_MIM]PF_6_. The maximum emission wavelength of 4-t-OP was 302.0 nm, while the maximum absorption wavelength of [C_8_MIM]PF_6_ was 212.0 nm, demonstrating that no overlap phenomenon of emission spectrum of the donor and absorption spectrum of the acceptor occurred. Therefore, it can be inferred that the fluorescence quenching of 4-t-OP by the four RTILs does not result from FRET phenomenon.

### Effect of RTILs Optical Property

The previous investigation has proved that all imidazolium ionic liquids exhibit an excitation wavelength-dependent fluorescence behavior [9, 36, 37]. In this experiment, we observed that the maximum excitation wavelength of the [C_8_MIM]PF_6_ was about 212.0 nm, and that the emission band of them ranged from 290.0 to 320.0 nm. The general emission characteristics of the four RTILs are quite similar. Under the conditions of the maximum excitation wavelength (227.0 nm) of 4-t-OP, Obviously, the FI of 4-t-OP/[C_8_MIM]PF_6_ complexes is far greater than that of the sole [C_8_MIM]PF_6_. Thus, the optical properties of the four RTILs hardly affect the fluorescent emission of 4-t-OP when they are used as the solvents.

### Evaluation of Analytical Performance

For the purpose of quantitative analysis, application of RTILs as solvent for spectrofluorimetric detection of 4-t-OP was further investigated. Herein, we chose 0.6 mM [C_8_MIM]PF_6_ in acetonitrile as an example to demonstrate selectivity and precision of this potential analytical method. A series of experiments were designed to evaluate the parameters including linearity, reproducibility, limit of detection and other characteristics of this method. The calibration curve was y = 479.8x + 90.756, the linearity range was 0.01–0.8 mg/L, and the correlation coefficient (R^2^) was 0.9990. The detection limits (at S/N = 3) of 4-t-OP was 3.7 μg/L. The relative standard deviation (RSD) of 4-t-OP (100 μg/L, *n* = 6) was 0.08 % for intra-day precision and 0.19 % for inter-day precision.

## Conclusions

The four RTILs, [C_8_MIM]BF_4,_ [C_14_MIM]BF_4,_ [C_8_MIM]PF_6_ and [C_14_MIM]PF_6_, had significantly fluorescence-quenching effects on the FIs of 4-t-OP. The quenching effect of 4-t-OP was decreased with increasing RTIL concentration (0.2–1.0 mM). However, no obvious hypsochromic or bathochromic phenomenon of the emission maxima was observed in the quenching process. The quenching effects by ionic liquids can rule out forming possibility of ground-state complex between the fluorophores in 4-t-OP and quencher (ionic liquids). The fluorescence quenching of 4-t-OP in [CnMIM]BF_4_ was greater than [CnMIM]PF_6_, but the carbon chain length on imidazolium ring had no significant relationship with the FI of 4-t-OP. Both the bimolecular quenching constant Ksv, estimated from S-V equation to be larger than 1.0 × 10^3^ L/mol, and the different temperature effects suggested that the fluorescence quenching of 4-t-OP by four RTILs was the simultaneous presence of dynamic and static quenching mechanism. The FI of DNSCl increased nearly 5-fold as compared to the control within [C_8_MIM]BF_4_, showing a sensitizing effect on the strong fluorescent chemicals. The mechanism of the different RTIL fluorescence-mediating effects on DNSCl and 4-t-OP was unclear. Because no overlap phenomenon of emission spectrum of 4-t-OP and absorption spectrum of [C_8_MIM]PF_6_ was observed, the fluorescence quenching of 4-t-OP by the four RTILs did not belong to FRET phenomenon. The FI of 4-t-OP/[C_8_MIM]PF_6_ complex is far greater than that of the sole [C_8_MIM]PF_6_, demonstrating that the optical properties of RTILs hardly affect the fluorescent emission of 4-t-OP. As an example of analytical performance, when 0.6 mM [C_8_MIM]PF_6_ in acetonitrile was used as the solvent, the detection limit of 4-t-OP was 3.7 μg/L, and the linearity range was 0.01–0.8 mg/L (R^2^ =0.9990). Overall, these results provide a theoretical foundation for application of RTILs in weak fluorescent chemicals.
